# Tunable Spin–Orbit Splitting in Bilayer Graphene/WSe_2_ Quantum Devices

**DOI:** 10.1021/acs.nanolett.5c02309

**Published:** 2025-08-07

**Authors:** Jonas D. Gerber, Efe Ersoy, Michele Masseroni, Markus Niese, Michael Laumer, Artem O. Denisov, Hadrien Duprez, Wister Wei Huang, Christoph Adam, Lara Ostertag, Chuyao Tong, Takashi Taniguchi, Kenji Watanabe, Vladimir I. Fal’ko, Thomas Ihn, Klaus Ensslin, Angelika Knothe

**Affiliations:** † Solid State Physics Laboratory, 27219 ETH Zürich, 8093 Zürich, Switzerland; ‡ Institut für Theoretische Physik, 9147 Universität Regensburg, D-93040 Regensburg, Germany; § Research Center for Materials Nanoarchitectonics, 52747 National Institute for Materials Science, 1-1 Namiki, Tsukuba 305-0044, Japan; ∥ Research Center for Electronic and Optical Materials, National Institute for Materials Science, 1-1 Namiki, Tsukuba 305-0044, Japan; ⊥ National Graphene Institute, 5292 University of Manchester, Manchester M13 9PL, U.K.

**Keywords:** graphene, transition metal dichalcogenides, proximity effect, spin−orbit coupling, quantum dot, quantum point contact

## Abstract

Bilayer graphene (BLG)-based quantum devices represent a promising platform for emerging technologies, such as quantum computing and spintronics. However, their intrinsically weak spin–orbit coupling (SOC) complicates spin and valley manipulation. Integrating BLG with transition metal dichalcogenides (TMDs) enhances the SOC via proximity effects. While this enhancement has been demonstrated in 2D-layered structures, 1D and 0D nanostructures in BLG/TMD remain unrealized, with open questions regarding SOC strength and tunability. Here, we investigate quantum point contacts and quantum dots in two BLG/WSe_2_ heterostructures with different stacking orders. Across multiple devices, we reproducibly demonstrate spin–orbit splitting up to 1.5 meV—more than 1 order of magnitude higher than in pristine BLG. Furthermore, we show that the induced SOC can be tuned in situ from its maximum value to near-complete suppression via the perpendicular electric field. This enhancement and in situ tunability establish the SOC as a control mechanism for dynamic spin and valley manipulation.

## Graphical abstract

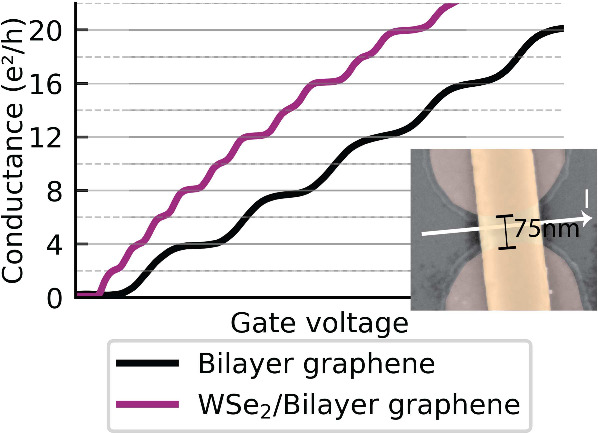

With its tunable band gap,^1^ high carrier mobility,^2^ and a nuclear-spin-free environment,^3^ bilayer graphene (BLG) is a promising material for advanced quantum devices in spintronics, valleytronics, and quantum computation. Recent experiments have demonstrated long spin and valley relaxation times in BLG-based quantum dots,^4–8^ underscoring its potential for spin qubits. For these applications, strong spin–orbit coupling (SOC) can be advantageous, enabling efficient spin control via electric dipole spin resonance^9^ and playing a key role in spin- and valleytronics, including spin–orbit valves^10^ and spin–valley filters.^11,12^ However, in pristine BLG, SOC is intrinsically weak, with a spin–orbit splitting (Δ_SO_) of only 40–80 μeV,^13–17^ arising from Kane–Mele^18^ and Bychkov–Rashba mechanisms.^19^ Furthermore, its limited in situ tunability^14,15^ poses additional challenges.

A promising approach to enhance SOC in BLG-based quantum devices is proximity coupling to TMDs with strong intrinsic SOC. This combination has been shown to significantly increase SOC in two-dimensional bulk BLG, resulting in spin–orbit splittings of up to several meV.^10,20–37^ Experimental estimates of SOC strength in BLG/TMD heterostructures were first obtained using traditional transport techniques, such as weak antilocalization measurements^24–28,36^ and Shubnikov–de-Haas oscillations.^30–32,34,35^

Unlike transport techniques, extracting SOC from confined quantum devices such as quantum dots (QDs) and quantum point contacts (QPCs) is more direct, model-independent, and less affected by disorder, allowing the precise determination of Δ_SO_. These devices probe energy gaps near the band edge, a regime typically inaccessible in 2D transport measurements. QPCs serve as a robust tool for detecting degeneracy lifting, while QDs enable precise spin–orbit gap measurements. Together, they provide a comprehensive view of spin–orbit effects, with their sensitivity to layer polarization enabling layer-resolved probing.

This work presents high-precision Δ_SO_ measurements in two BLG/WSe_2_ heterostructures using QPCs and QDs. We identify the spin–valley—Zeeman SOC (also referred to as the Ising SOC in the literature) as the dominant mechanism causing this splitting. Additionally, we demonstrate quantized conductance and a Coulomb blockade in BLG/TMD systems. Our experimental data closely align with single-particle calculations, indicating a strong understanding of electronic states in confined BLG/TMD structures. By systematically varying the displacement field in devices with different stacking order types, we achieve in situ tuning of spin–orbit splitting over more than an order of magnitude. This tunability is essential for developing adaptive and flexible quantum devices for applications in quantum computing, spintronics, and valleytronics.

Using the gate structure shown in the false-color SEM top view (Figure 1a), we electrostatically define QDs and QPCs. The two split gates (SGs), separated by a lithographic width of 75 nm, work in combination with the graphite back gate to open a band gap and create a 1D confinement in bilayer graphene. A channel gate (CG), positioned above the SGs and insulated by a layer of Al_2_O_3_, controls the local electrostatic potential within the channel. By tuning the channel gate voltage (*V*_CG_), we can define either QPCs or QDs. Notably, QDs in this design rely on pn-junction tunneling barriers, allowing the formation of only p-type dots with n-type leads and vice versa.^38,39^ A cross-sectional view of the heterostructure is shown in Figure 1b. In this study, we investigate quantum devices in two heterostructures: WSe_2_-on-BLG (sample A, with a WSe_2_/BLG twist angle of 0 ± 2°) and BLG-on-WSe_2_ (sample B, 4 ± 2° twist angle). Details on sample fabrication and twist angle determination are provided in SI Section A.

**Figure 1:**
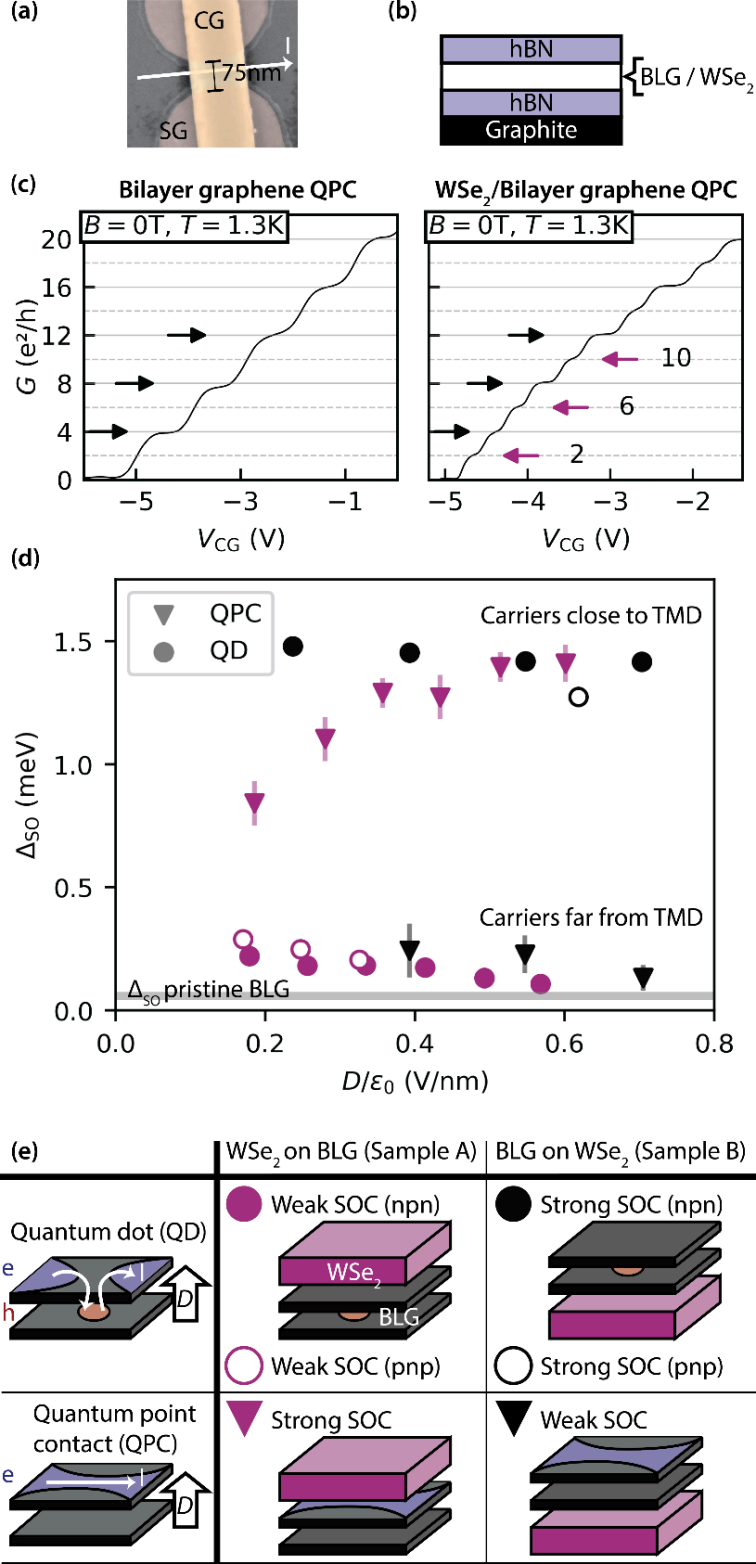
(a) False-color SEM image showing the top view of the measured devices. (b) Schematic cross-sectional view of the BLG/TMD heterostructures. (c) Quantized conductance of QPCs in pristine BLG and BLG/WSe_2_, corrected for parasitic resistances (see Supporting Information (SI) Section B2). Black arrows indicate 4-fold-degenerate plateaus, while the magenta arrows highlight SOC-induced degeneracy lifting. (d) Extracted spin–orbit splitting of two heterostructures (magenta/black) as a function of displacement field applied beneath the split gates. The gray band marks the typical range of Δ_SO_ observed in pristine BLG quantum devices. (e) Data exhibiting excellent agreement with expectations based on layer polarization. Open symbols correspond to measurements taken with reversed displacement field polarity relative to the convention defined in panel e.

As the first compelling evidence of enhanced proximity-induced SOC, Figure 1c compares the quantized conductance of a QPC in a pristine BLG with that in a WSe_2_/BLG heterostructure. Both measurements were performed at a temperature of *T* = 1.3 K and zero magnetic field, with corrections applied for parasitic resistances as detailed in SI Section B2. The pristine BLG QPC shows quantized conductance steps in units of 4*e* ^2^/*h*, reflecting the 4-fold degeneracy of its energy levels. This behavior arises because the intrinsic Δ_SO_ is smaller than the thermal energy *k*_B_ *T*, consistent with previous studies.^14,40–43^ In contrast, the WSe_2_/BLG heterostructure exhibits conductance steps in units of 2*e* ^2^/*h* (magenta arrows in Figure 1c), indicating that the 4-fold degeneracy has been lifted into two pairs (2 + 2), with a splitting energy significantly exceeding *k*_B_ *T*.

Figure 1d presents the central result of this work: a summary of the experimentally extracted spin–orbit splittings for the first modes in all measured QPC devices (triangles) and the first single-particle levels in all QD devices (circles). All measurements were conducted at a base temperature of 10 mK, with the extraction procedure detailed in subsequent chapters. Figure 1e provides a legend of Figure 1d, indicating the stacking order of the two heterostructures: sample A (purple symbols) and sample B (black symbols).

We begin by discussing measurements taken at a displacement field that yields n-type QD leads and n-type QPC channels, corresponding to carrier polarization in the top layer of the BLG (indicated in blue in Figure 1e). The layer polarization of carriers^44^ plays a critical role: in sample A, where carriers reside in the layer adjacent to the WSe_2_, the QPC exhibits a large Δ_SO_ (filled purple triangles in Figure 1d). In contrast, in sample B, where the carriers occupy the remote layer, Δ_SO_ is significantly reduced (filled black triangles). A complementary trend is observed in the QD measurements. In sample A, the p-type QD forms in the layer remote from the WSe_2_ layer, resulting in a small Δ_SO_ value (filled purple circles). Conversely, in sample B, the QD forms adjacent to the WSe_2_, leading to a significantly larger Δ_SO_ (filled black circles).

Reversing the sign of the displacement field results in p-type leads with holes once again polarized in the top layer. Consequently, for an n-type quantum dot, carriers remain polarized in the bottom layer in both samples A and B. As a result, the QD in sample A continues to exhibit weak SOC (open purple circles in Figure 1d), while the QD in sample B maintains strong SOC (open black circles). The layer polarization can be continuously tuned in all devices, enabling modulation of Δ_SO_ as a function of the displacement field applied beneath the split gates, as shown in Figure 1d. As the displacement field decreases, the layer polarization is reduced, leading to a more symmetric distribution of the electronic wave function across both layers. This results in convergence of SOC values between the strong and weak SOC regimes. This trend is clearly observed in both QDs and QPCs in sample A (magenta symbols). For sample B, data at low-displacement fields are absent due to the loss of quantum confinement. Minor Δ_SO_ differences between the data sets arise from strain or twist-angle variations between samples.^21,22,32,45,46^ In addition, slight sample-to-sample variations in the effective displacement field exist, as discussed in SI Section B5.

Owing to the similarly low twist angles in samples A and B, the measured SOC values are consistent with recent transport experiments in BLG/WSe_2_ heterostructures.^32^ At large displacement fields, the enhanced spin–orbit splitting Δ_SO_ saturates between 1.3 and 1.5 meV. By switching the layer polarization in situ, Δ_SO_ can be tuned down to 100–300 μeV. Both the maximum and minimum values significantly exceed the typical Δ_SO_ in pristine BLG quantum devices—indicated by the gray band in Figure 1d—demonstrating the additive effect of proximity-induced SOC on top of the intrinsic Kane–Mele SOC in BLG. Moreover, the observed in situ tunability of Δ_SO_ via layer polarization aligns with the expected short-range nature of the orbital overlap mechanism responsible for proximity-induced SOC.^20,36^ While WSe_2_ is commonly used to induce enhanced SOC in BLG, other TMDs can produce a comparable increase in Δ_SO_, as demonstrated by data from a MoS_2_/BLG quantum device presented in SI Section E2.

This section focuses on characterizing proximity-induced SOC in quantum dot devices. We begin with bias spectroscopy measurements on a npn-QD in sample B (BLG/WSe_2_), where strong SOC is expected due to the proximity of the QD to the TMD layer (Figure 2a). The quantum dot potential is tuned via the channel gate voltage *V*_CG_. Fintie-bias spectroscopy at the 0 h →1 h transition (Figure 2b) reveals a clear spin–orbit splitting between the ground and first excited states, with Δ_SO_ = 1.42 ± 0.02 meV. This value corresponds to the black circle in Figure 1d at a displacement field of *D*/ϵ_0_ = 0.7 V/nm.

**Figure 2:**
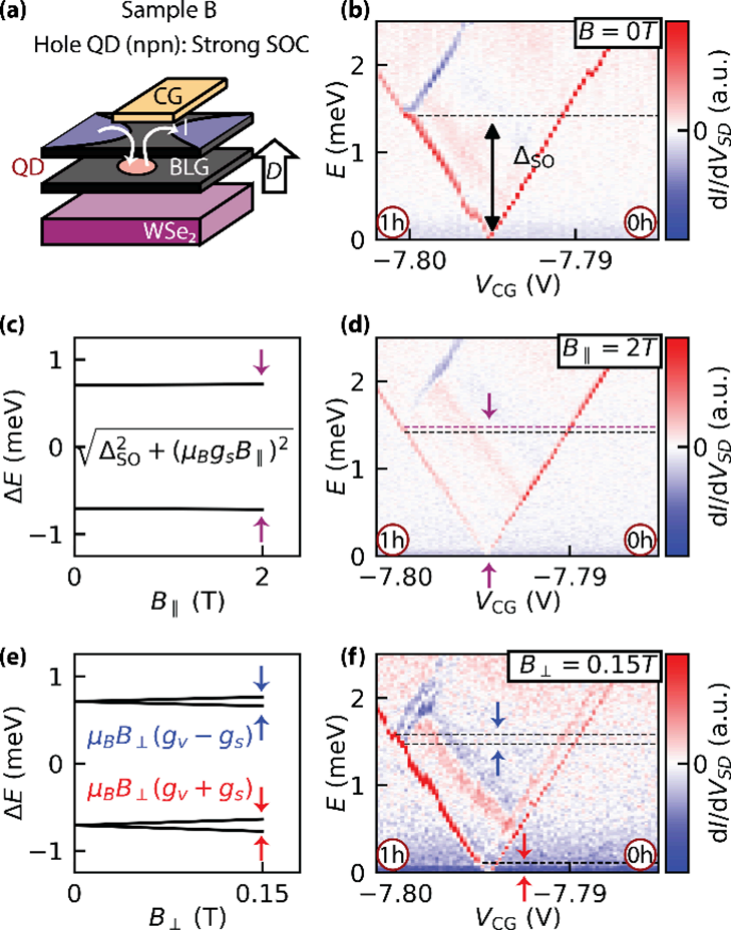
(a) Schematic of the measured npn-type QD in the BLG/WSe_2_ sample. (b) Finite-bias spectroscopy at the 0 h →1 h charge transition at zero magnetic field. The intersection of the excited state line with the Coulomb diamond edges (black dashed line) yields a spin–orbit gap of Δ_SO_ = 1.42 ± 0.02 meV. (c) Calculated evolution of quantum states in an in-plane magnetic field using the extracted Δ_SO_. (d) Corresponding finite-bias measurement at *B*_∥_ = 2 T. The excited-state energy (magenta dashed line) shows only a minor shift relative to the zero-field gap (black dashed line). (e) Calculated energy evolution in a perpendicular magnetic field using Δ_SO_ and *g*_*v*_ = 14, predicting the characteristic splitting of the two Kramers pairs. (f) Experimental finite-bias measurement at *B*_⊥_ = 0.15 T, in agreement with the predicted splitting.

To verify that the observed excited state corresponds to the spin–orbit split-off Kramers pair, we investigated its evolution under an in-plane magnetic field (*B*_∥_). In this confined QD regime, Rashba-type spin–orbit coupling is expected to be strongly suppressed compared to the 2D case.^47^ Under the assumption of spin–valley—Zeeman SOC (see SI Section C for details) as the dominant mechanism, the expected energy splitting follows $\Delta E=\sqrt{{\Delta_{\mathrm{SO}}}^{2}+{\left(g_{S}\mu_{B}B_{∥}\right)}^{2}}$ , as illustrated in Figure 2c. At *B*_∥_ = 2 T, this model predicts a modest increase of 19 μeV relative to the zero-field value. In the experiment (Figure 2d), the measured splitting at *B*_∥_ = 2 T (magenta dashed line) shows a small increase of 60 ± 30 μeV compared to the zero-field spin–orbit gap (black dashed line), consistent with expectations. The discrepancy is attributed to a slight perpendicular field component due to an unavoidable sample tilt. Importantly, the observed field dependence is far weaker than that expected from a linear spin–Zeeman splitting with *g*_S_ = 2, which would result in a shift of 230 μeV.

Additional confirmation of the spin–orbit nature is obtained by applying a perpendicular magnetic field (*B*_⊥_). Assuming predominantly out-of-plane SOC and using the previously extracted values Δ_SO_ = 1.42 ± 0.02 meV and *g*_v_ ≈ 14, we predict the characteristic magnetic-field evolution of the Kramers pairs shown in Figure 2e. This prediction is consistent with the finite-bias spectroscopy data presented in Figure 2f. In this device, increasing *B*_⊥_ significantly suppressed the transport current, which limits the precision of extracted *g*_*v*_. Nonetheless, the magnetic field dependence observed across measurements aligns well with a single-particle model incorporating proximity-induced SOC, supporting the identification of the excited state as a spin–orbit split-off level. The full evolution of the QD states for this sample is provided in SI Section E5.

We now turn to the analysis of the QPC in sample A (BLG/WSe_2_ heterostructure, Figure 3a) to extract both the magnitude and the nature of the SOC. Figure 3b presents the calculated single-particle band structure of a 30 nm wide QPC in BLG/WSe_2_, based on density functional theory (DFT) SOC parameters from ref 33. The lowest-energy subband, formed by the K^+^ ↑/K^–^ ↓ states, is separated by the spin–orbit gap Δ_SO_ from the K^+^ ↓/K^–^ ↑ states.

**Figure 3:**
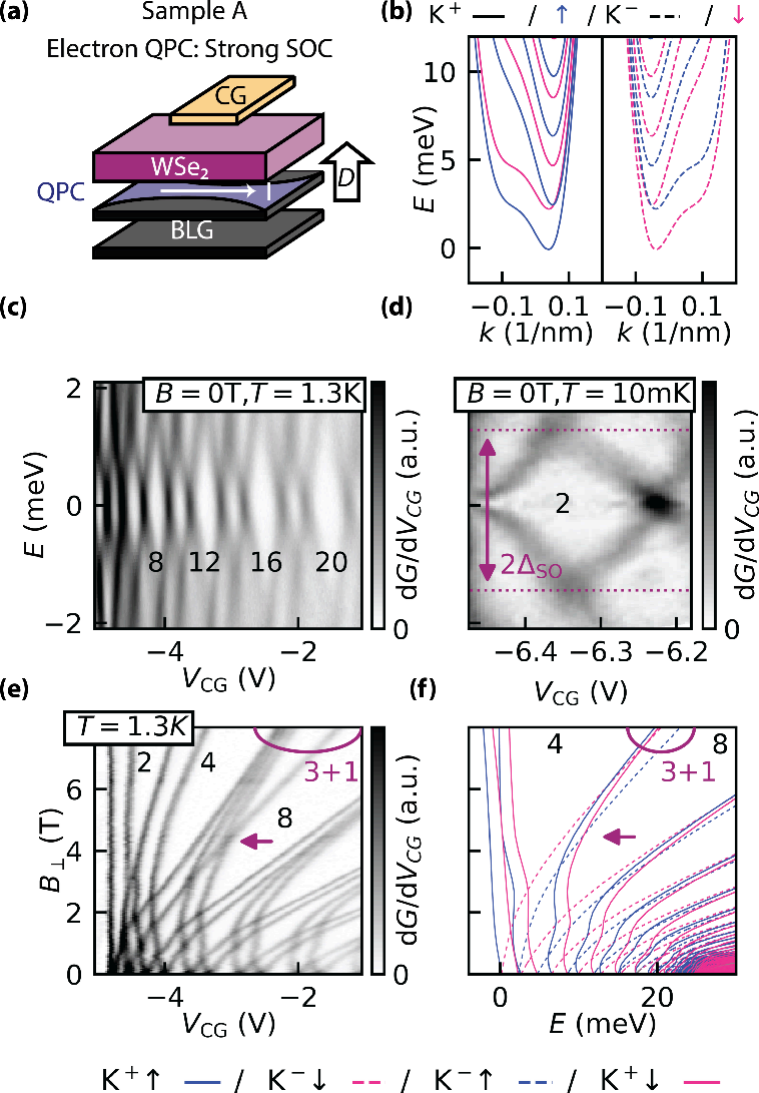
(a) Schematic of the electron QPC in the WSe_2_/BLG heterostructure. (b) Single-particle band structure of a 30 nm wide QPC on BLG/WSe_2_, calculated using the DFT SOC parameters from ref 33. (c) Bias spectroscopy of the QPC at *B* = 0 and *T* = 1.3 K. The alternating white diamond-shaped regions correspond to conductance plateaus, with values indicated in units of *e* ^2^/*h*. (d) High-resolution bias spectroscopy of the first *G* = 2*e* ^2^/*h* plateau, measured at 10 mK. The diamond height directly yields 2Δ_SO_. (e) Transconductance d*G*/d*V*_CG_ as a function of *V*_CG_ and *B*_⊥_, measured at *T* = 1.3 K. Black labels indicate corresponding conductance quantum numbers. (f) Calculated single-particle magnetic subband evolution for a 30 nm wide QPC in BLG/WSe_2_, showing excellent agreement with the experimental data across both low and high magnetic fields. The characteristic “3 + 1” feature, resulting from spin–valley—Zeeman coupling, is highlighted with the magenta circle. The magenta arrow marks the crossing between the 1K^–^ ↑ – 3K^+^ ↓ states, in panels e and f. This “3 + 1” feature is discussed more in detail in SI Section E6.

This gap is experimentally determined by using bias spectroscopy (Figure 3c). The black numbers indicate the conductance values in units of *e* ^2^/*h*. The corresponding zero-bias conductance trace *G*(*V*_CG_) is shown in the right panel of Figure 1c. To enhance energy resolution, we remeasured the first spin–orbit split plateau at 2*e* ^2^/*h* in a dilution refrigerator with a base temperature of 10 mK (Figure 3d). The height of the corresponding diamond directly yields 2Δ_SO_, from which we extract Δ_SO_ = 1.37 ± 0.08 meV. This data point corresponds to the magenta triangle in Figure 1d at *D* /ϵ_0_= 0.6 V/nm. SI Section B4 describes the procedure used to determine the corresponding energy scale.

To characterize the quantum states, we analyzed the magnetic depopulation of magnetoelectric subbands by measuring d*G*/d*V*_CG_ as a function of *B*_⊥_ (Figure 3e). The conductance steps appear as dark lines, each of which splits into two at low *B*_⊥_, consistent with the valley–Zeeman effect. This pronounced splitting confirms that the degenerate states at *B* = 0 originate from opposite valley flavors. As the field increases, lines corresponding to states with the same valley and subband index evolve in parallel but remain offset due to spin–orbit splitting. While the spin–Zeeman effect is unresolved at low magnetic fields, opposite spin flavors lead to the formation of a “3 + 1” feature at high magnetic fields, as discussed in SI Section E6. Unlike that observed in pristine BLG (see SI Section E3), the enhanced spin–orbit coupling combined with the valley–Zeeman effect enables all four states to be individually resolved.

To further elucidate the interplay between subband spacing and SOC, we compare the measured spectrum (Figure 3e) and single-particle calculations (Figure 3f). The modeling of the electrostatically defined QPC in bilayer graphene follows previous approaches for pristine BLG,^42,43,48^ with additional details provided in SI Section C. Using the Hamiltonian outlined therein, we computed the QPC subband spectrum shown in Figure 3f. The calculations use the DFT SOC parameters for BLG/WSe_2_ from ref 33 and a channel width of *L* = 30 nm, estimated from the measured level spacing in Figure 3e. The discrepancy between this value and the lithographically defined width (*L* = 75 nm) arises from stray electric fields near the split gates, which effectively narrow the electronic channel. The agreement between the measured subband evolution and the theoretical spectrum is excellent across both low and high magnetic fields. Importantly, this agreement is sensitively dependent on the choice of SOC parameters in the model. We do not reproduce the measured magnetic field pattern for parameters strongly deviating from the DFT parameters of ref 33. In SI Section D, we explore the influence of various SOC parameters and show that Rashba-type SOC has a minimal impact on the magnetic field dependence of the subbands. These findings confirm that the observed Δ_SO_ predominantly arises from proximity-induced spin–valley—Zeeman SOC.

In this section, we examine the magnetic subband evolution in greater detail and present evidence for an exchange-enhanced *g*-factor, demonstrating that this platform is well-suited for exploring many-body physics. We remeasure the QPC in sample A (WSe_2_/BLG, Figure 4a) at a temperature of 10 mK and compare its magneto-transconductance to that of a second QPC fabricated on the same sample (Figure 4b). In both plots, the evolution of the first subband states is color-highlighted, with the subband assignment confirmed by high-field measurements (SI Section E6). Despite identical lithographic widths, the subband spectra in Figure 4a,b differ significantly. In particular, the subband spacing in Figure 4a is markedly larger than that in Figure 4b, arising from a higher applied split-gate voltage in Figure 4a and possible device-to-device variations of the electrostatic landscape.

**Figure 4:**
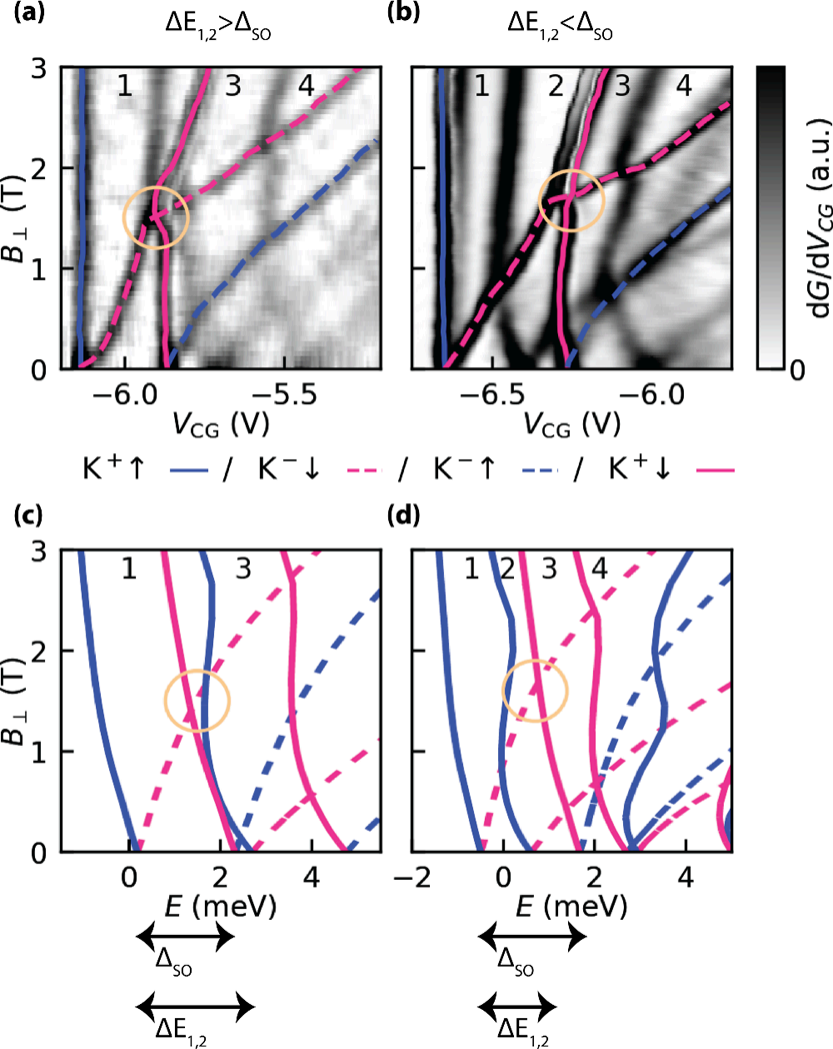
(a, b) Experimentally measured transconductance versus *B*_⊥_ of two QPCs in a WSe_2_/BLG heterostructure (sample A) at 10 mK. The left QPC (a) is the same device shown in Figure 3. The right QPC (b) is located on the same heterostructure stack but exhibits a different subband spectrum, likely due to differences in applied gate voltages and possible device-to-device variations. (c, d) Single-particle calculations of QPC energy levels in a BLG/WSe_2_ heterostructure using DFT SOC parameters from ref 33 with a QPC width of *L* = 30 nm (left) and *L* = 50 nm (right). The measured subband evolution, with the states of the first subband highlighted, shows good agreement with theoretical calculations. A notable deviation from the theoretical prediction is marked by an orange circle, indicating a strong bending of the 1K^–^ ↓ mode when crossing the 1K^+^ ↓ mode.

To interpret the experimental observations, we compare the data to single-particle calculations for QPCs with different channel widths, adjusted to reproduce the measured subband spacings while keeping the SOC parameters fixed. In Figure 4c, for a channel width of 30 nm, the calculated subband spacing Δ*E*_1,2_ exceeds the spin–orbit-induced splitting Δ_SO_ and vice versa for the 50 nm wide QPC (Figure 4d).

A prominent deviation between the experimental data and single-particle calculations is the pronounced nonlinearity observed in the magnetic field evolution of the subbands. In both measured QPCs the 1K^–^ ↓ subband exhibits a pronounced bending after crossing the 1K^+^ ↓ state, as highlighted by the orange circle. We attribute this feature to an enhancement of the effective *g*-factor, g*, arising from exchange interactions. Enhancements of g* have been reported in various low-dimensional systems^49–51^ and are commonly attributed to spin-based exchange interactions.^52,53^ In our case, however, the crossing between the 1K^–^↓ and 1K^+^ ↓ states involves a change in the valley occupancy. This suggests that interaction effects in our system are caused by more complex spin–valley interactions.

While a complete theoretical description of the effect is beyond the scope of this work, our results highlight the interplay of spin, valley, and subband index in shaping the magnetic response of QPCs in graphene-based heterostructures. Owing to the resolution of individual subbands, such behavior becomes experimentally accessible in BLG/TMD systems, establishing them as a promising platform for future investigations of correlated electronic phenomena.

We have presented direct energy spectroscopy measurements of tunable spin–valley—Zeeman spin–orbit coupling in QPCs and QDs based on BLG/WSe_2_ heterostructures. Our results demonstrate that the SOC in BLG can be enhanced from below 80 μeV to 1.5 meV via proximity to a TMD layer. This strong SOC regime, reached at large displacement fields, remains robust in the range of 1.3–1.5 meV across device types (QPCs and QDs) and carrier polarities (electrons and holes), establishing BLG/TMD heterostructures as a versatile platform for engineering strong SOC. We further show that the spin–orbit gap Δ_SO_ is highly tunable—by over an order of magnitude—via the displacement field, which controls the layer polarization in BLG. This tunability opens the door to QD architectures with multiple gates, such as those demonstrated in refs 38 and 39 , that can switch between strong-SOC (nn′n) and weak-SOC (npn) QD regimes in situ, without reversing the displacement field. The ability to modulate SOC within a single device allows a direct comparison of quantum phenomena under distinct SOC conditions, effectively adding a new dimension of control. While our measurements primarily reveal a spin–valley—Zeeman SOC, the detection and fine control of Rashba SOC in these confined hybrid systems remain an open question for future investigation.

Our experimental results show excellent agreement with single-particle modeling across a broad range of parameters. Given the full lifting of degeneracies in the subband spectra, remaining deviations can be attributed to many-body interactions, indicating that this platform is well-suited for exploring correlated phenomena.

The combination of high material quality, gate-tunable confinement, and proximity-induced SOC makes BLG/WSe_2_ heterostructures compelling candidates for next-generation spintronic, valleytronic, and quantum information devices. In particular, the ability to switch between strong and weak SOC regimes in situ provides a powerful lever for tailoring and optimizing device functionality.

## Supplementary Material



## Notes

Details on device fabrication, measurement setup, data analysis, model details, and additional measurement data (PDF)
