# Supplementary material for: Tunable Spin–Orbit Splitting in Bilayer Graphene/WSe2 Quantum Devices
Source: Nano Lett. 2025 Aug 7;25(33):12480–6. doi: 10.1021/acs.nanolett.5c02309 (PMC12371879; doi:10.1021/acs.nanolett.5c02309)
Supplement: Supplementary file 1 [file nl5c02309_si_001.pdf]

Supporting Information

Tunable spin–orbit splitting in bilayer graphene/WSe_2 quantum devices

Jonas D. Gerber[1,*], Efe Ersoy[1], Michele Masseroni[1], Markus Niese[1], Michael Laumer[2], Artem O. Denisov[1], Hadrien Duprez[1], Wister Wei Huang[1], Christoph Adam[1], Lara Ostertag[1], Chuyao Tong[1], Takashi Taniguchi[3], Kenji Watanabe[4], Vladimir I. Fal'ko[5], Thomas Ihn[1], Klaus Ensslin[1], Angelika Knothe[2]

[1]*Solid State Physics Laboratory, ETH Zürich, 8093 Zürich, Switzerland*
[2]*Institut für Theoretische Physik, Universität Regensburg, D-93040 Regensburg, Germany*
[3]*Research Center for Materials Nanoarchitectonics, National Institute for Materials Science, 1-1 Namiki, Tsukuba 305-0044, Japan*
[4]*Research Center for Electronic and Optical Materials, National Institute for Materials Science, 1-1 Namiki, Tsukuba 305-0044, Japan*
[5]*National Graphene Institute, University of Manchester, Manchester M13 9PL, UK*
*Email: gerberjo@phys.ethz.ch

Appendix A: Device fabrication

The 2D materials used in this study were prepared by mechanical exfoliation of bulk crystals. For the WSe_2 and MoS_2 layers, we used material from *HQ graphene*. The corresponding heterostructures were assembled using a polymer-based dry transfer technique. The stacks presented in the main text have the following layer thicknesses.

Layer Nr	WSe_2-on-BLG (A)	BLG-on-WSe_2 (B)
1	hBN: 21 nm	hBN: 23 nm
2	WSe_2: 5 layers	BLG
3	BLG	WSe_2: 3 layers
4	hBN: 85 nm	hBN: 42 nm
5	Graphite: 3 nm	Graphite: 25 nm

TABLE A.1. Layer composition and thicknesses from top to bottom for samples A and B.

Using atomic force microscopy (AFM), the relative twist angle between WSe_2 and BLG was determined to be $(0 \pm 2)^\circ$ for the WSe_2-on-BLG stack (sample A) and $(4 \pm 2)^\circ$ for the BLG-on-WSe_2 heterostructure (sample B). It is important to note that exfoliated edges in hexagonal materials predominantly exhibit zig-zag or armchair orientations, leading to an intrinsic $0^\circ/30^\circ$ uncertainty. Since we observe a significant enhancement of the spin–orbit gap Δ_{SO}, we conclude that the twist angle in both devices is close to 0°. This is supported by both theoretical [1–4] and experimental [5] studies, which indicate a strong reduction of λ_{VZ} near 30°.

We used standard electron-beam lithography (EBL) and metal deposition techniques for device fabrication. 3/20 nm Cr/Au split gates (SGs) were formed first, creating a 75 nm wide channel. Ohmic contacts were formed by a CHF_3/O_2 etch followed by 5/50 nm Cr/Au metal deposition. Finally, a 200 nm wide and 10/90 nm high Cr/Au channel gate is deposited on top of a 20 nm ALD grown Al_2O_3 layer.

Appendix B: Measurement setup and Data Analysis

1. Measurement setup

Measurements were conducted both in a variable temperature insert (1.3 K base temperature) and a dilution refrigerator (10 mK base temperature). We use the measurement setup shown in Fig. B.1.

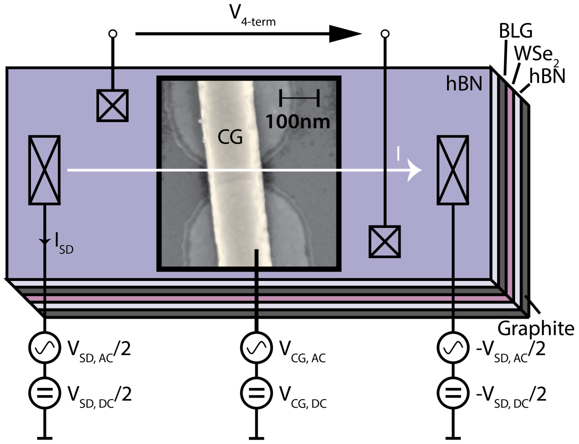

FIG. B.1. Measurement setup using an AC and DC voltage applied source-drain bias, measuring the current. A DC voltage is applied to the channel gate with the option of additionally applying an AC bias. For QPC measurement, the setup allows four-terminal voltage measurements.

A small AC voltage on a DC voltage was applied to the source-drain (SD) leads. The resulting current I_{SD} (referred to as I) was measured using an IV converter. The conductance shown throughout this work was obtained from the differential current response. Unless otherwise

stated, all data presented in the figures were acquired using this AC measurement technique.

This lock-in technique reduces noise, which is crucial for accurately extracting excited state energies. All initial measurements were performed in a two-terminal configuration. In high-resistance measurements, such as in QDs, this method introduces a small, universal offset in dI/dV_{SD}, which depends on $V_{\mathrm{SD,AC}}$ and $f_{\mathrm{SD,AC}}$. We correct for this offset in Figs. 2b,d,f and Figs. E.5b,c,d. Moreover, this two-terminal setup was used for QPC measurements at $T = 1.3\,\mathrm{K}$ (Figs. 3c,e), where the line resistances of roughly $100\,\Omega$ are negligible.

Additional contacts on the sample allow for a four-terminal voltage $V_{4-\mathrm{term}}$ measurement. This four-terminal setup was used for the $T = 10\,\mathrm{mK}$ transconductance measurements (Figs. 4a,b) to eliminate artifacts from the $10\,\mathrm{k}\Omega$ filtering resistances at these low-temperatures. To present accurate conductance values for the conductance traces in Fig. 1c, we further correct for parasitic resistances, as discussed in Appendix B 2.

For the QPC bias spectroscopy (Figs. 3c,d), an additional AC voltage was applied to the channel gate, superimposed on the DC voltage. This approach optimizes the measurement of transconductance (dG/dV_{CG}), as explained in more detail in Appendix B 3.

2. Correcting for parasitic resistances in QPCs

In this chapter, we discuss the correction for parasitic resistances in order to recover the conductance plateaus of the QPCs.

Despite employing a four-terminal setup, the measured conductance, defined as $G_{\mathrm{raw}} = I/V_{4-\mathrm{term}}$, does not directly correspond to the intrinsic QPC conductance (G_{QPC}). This discrepancy arises because both the QPC and the ungated BLG contribute to a voltage drop between the four-terminal contacts. In the low-resistance regime of the QPC, the series conductance (G_{S}) of the ungated BLG is of a similar magnitude than the actual QPC conductance G_{QPC}. Consequently, G_{S} must be considered to obtain precise conductance values, particularly at high mode numbers.

Additionally, some devices have a parallel conductance path (G_{P}) alongside the QPC. If this parallel conductance is spatially separate from the channel and remains constant across all mode numbers, we can subtract it from the measurement. The intrinsic QPC conductance G_{QPC} is then obtained using the equivalent circuit shown in Fig. B.2a with the following correction formula:

$$G_{\mathrm{QPC}} = \frac{G_{\mathrm{raw}}(G_{\mathrm{S}} + G_{\mathrm{P}}) - G_{\mathrm{S}}G_{\mathrm{P}}}{G_{\mathrm{S}} - G_{\mathrm{raw}}}. \tag{B1}$$

By using these parameters G_{S} and G_{P}, we corrected the conductance traces in Fig. 1c by $G_{\mathrm{S}} = 600e^2/h$ and $G_{\mathrm{P}} = 8.5e^2/h$ for the BLG QPC and $G_{\mathrm{S}} = 39.1e^2/h$ and $G_{\mathrm{P}} = 6.75e^2/h$ for the WSe_2/BLG QPC. Figure B.2b illustrates this correction for the WSe_2/BLG QPC on sample A, where more than 10 conductance plateaus were recovered using just two parasitic resistance parameters, demonstrating the validity of the model. The data from this QPC, labeled QPC1, is the one shown in Fig. 1c, Figs. 3c,d,e, and Fig. 4a.

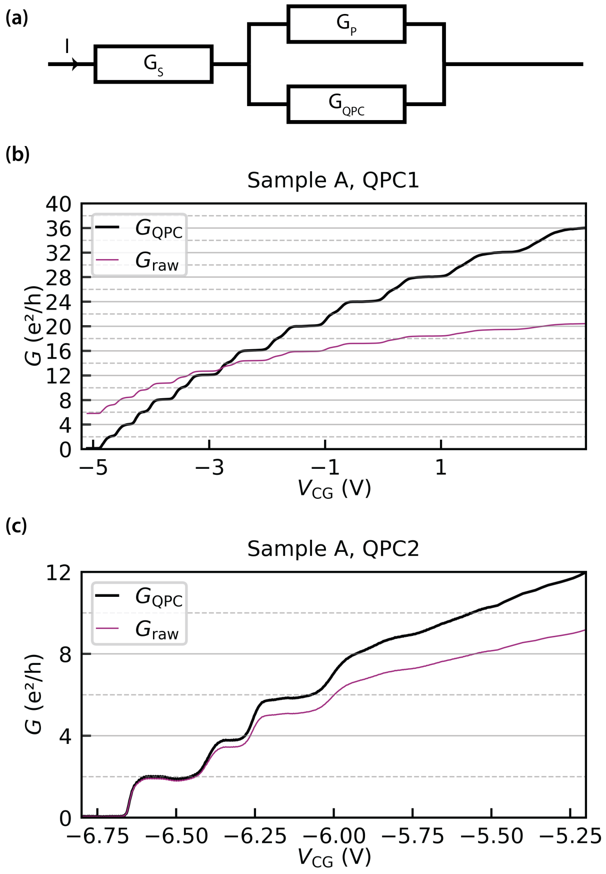

FIG. B.2. **(a)** Equivalent circuit illustrating the measured conductance $G_{\mathrm{raw}} = I/V_{4-\mathrm{term}}$ and its dependence on the parasitic resistances G_{S} and G_{P}. The correct QPC conductance G_{QPC} is extracted using these parameters. **(b)** Conductance of QPC1 (same as in the main study) showing both the uncorrected (G_{raw}) and corrected conductance (G_{QPC}) with $G_{\mathrm{S}} = 39.1e^2/h$ and $G_{\mathrm{P}} = 6.7e^2/h$ at $T = 1.3\,\mathrm{K}$ and $B = 0\,\mathrm{T}$. **(c)** G_{raw} and G_{QPC} of QPC2 on the same stack with $G_{\mathrm{S}} = 39.1e^2/h$ and $G_{\mathrm{P}} = 0$ at $T = 10\,\mathrm{mK}$ and $B = 0\,\mathrm{T}$.

The correctness of the extracted conductance plateaus is supported by the measured magnetic field pattern in Fig. 3c. For BLG (Appendix E 3), this magnetic field pattern resembles the ones in Refs. [6–10].

To further validate the correction procedure, we present additional data from a second QPC (QPC2, shown in Fig. B.2c and Fig. 4b) on the same WSe_2/BLG heterostructure (sample A) as QPC1. This QPC2 differs from QPC1 in that it does not possess a parallel conductance channel ($G_{\mathrm{P}} = 0$). Here, the two-fold de-

generacy lifting due to enhanced SOC is already evident in the raw data, with the lowest plateau occurring at $G_{\mathrm{QPC}} \approx G_{\mathrm{raw}} \approx 2e^2/h$. At higher mode numbers, applying the same series conductance correction ($G_{\mathrm{S}} = 39.1e^2/h$) as in QPC1 yields quantized conductance in steps of $2e^2/h$. Since QPC1 and QPC2 are connected in parallel within the same four-terminal contact configuration, they share the same ungated graphene leads in series, and thus the same G_{S}. This further validates that G_{S} is a global sample property, while G_{P} remains device-specific.

3. Transconductance measurement using two-frequency lock-in detection

The transconductance $dG/dV_{\mathrm{CG}} = d^2I/dV_{\mathrm{SD}}/dV_{\mathrm{CG}}$ in the V_{CG}-B plots (Fig. 3e and Figs. 4a,b) is computed numerically. This approach works well since data points used for differentiation are recorded sequentially.

In contrast, the measurement procedure for bias spectroscopy (Figs. 3c,d) differs, as here, we continuously sweep the source-drain voltage V_{SD} while stepping the channel gate voltage V_{CG}. Taking a numerical derivative in the V_{CG} direction in this case introduces significant sensitivity to noise and drift because data points are differentiated that were not recorded consecutively. To avoid these issues, we measure in a two-frequency lock-in amplifier setup, as illustrated in Fig. B.1, with a source-drain AC frequency of $f_{\mathrm{SD}} = 130\,\mathrm{Hz}$ and a channel gate frequency of $f_{\mathrm{CG}} = 35\,\mathrm{Hz}$. The resulting source-drain current I can be expressed as:

$$I(V_{\mathrm{SD}}, V_{\mathrm{SD}}) \approx I_0 + \frac{\partial I}{\partial V_{\mathrm{SD}}} dV_{\mathrm{SD}} + \frac{\partial I}{\partial V_{\mathrm{CG}}} dV_{\mathrm{CG}} + \frac{\partial^2 I}{\partial V_{\mathrm{SD}} \partial V_{\mathrm{CG}}} dV_{\mathrm{SD}} dV_{\mathrm{CG}} + \frac{\partial^2 I}{2\partial V_{\mathrm{SD}}^2} dV_{\mathrm{SD}}^2 + \frac{\partial^2 I}{2\partial V_{\mathrm{CG}}^2} dV_{\mathrm{CG}}^2 \quad \text{(B2)}$$

The first lock-in amplifier (f_{SD}), with a time constant of 10 ms, outputs a signal proportional to

$$\frac{\partial I}{\partial V_{\mathrm{SD}}} dV_{\mathrm{SD}} \quad \text{(B3)}$$

which remains constant over time, as well as

$$\frac{\partial^2 I}{\partial V_{\mathrm{SD}} \partial V_{\mathrm{CG}}} dV_{\mathrm{SD}} dV_{\mathrm{CG}}, \quad \text{(B4)}$$

which is modulated by the frequency f_{CG}. The second lock-in amplifier (f_{CG}) then directly extracts the transconductance dG/dV_{CG}.

4. Converting voltages into energy scales

For carrier transport through the high-resistance quantum dot, nearly the entire source-drain voltage drops across the dot. As a result, changes in source-drain voltage directly translate to changes in the quantum dot energy levels, such that $\Delta V_{\mathrm{SD}} = \Delta E/e$. The spin–orbit splitting is directly extracted from the intersection of the excited state line with the edge of the Coulomb diamond, fitted by two straight lines.

For the lower-resistive QPC, parasitic resistances must be considered, especially in the low-resistance regime. First, we determine the series resistance G_{S}, as shown in the previous subsection. The voltage drop across the QPC — and thus the corresponding energy is then obtained by correcting for the series resistance contribution using $\Delta E = e(V_{4-\mathrm{term}} - I/G_{\mathrm{s}})$. We extract Δ_{SO} by fitting the diamond edges with four individual lines and dividing its height by two.

5. Characterizing the displacement field

All displacement field values presented in the main text correspond to the field underneath the split gates in operation. This allows us to use the analytical simple plate capacitor model instead of accounting for the geometry of stray field components from the channel and split gates, which would be necessary for calculating potential landscape in the channel. Since the spin–orbit gap Δ_{SO} is extracted always for the first carrier/subband, the displacement field in the channel is close to pinch-off at the bend-edge and thus closely matches the value beneath the split gates.

Additionally, because the channel gate manipulates both the density and the displacement field during a measurement, the displacement field beneath the split gates provides a more consistent metric for comparison.

Its value is defined by:

$$D = 1/2\,(C_{\mathrm{B}} V_{\mathrm{BG}} - C_{\mathrm{T}} V_{\mathrm{SG}}) + D_0 \quad \text{(B5)}$$

with V_{BG} as back gate and V_{SG} as split gate voltage. To calculate the capacitances C_{T} and C_{B}, we use the thicknesses provided in Appendix A with $\epsilon_r = 3.24$ for hBN [11] and $\epsilon_r = 6$ for WSe_2 [12]. Due to the relatively large ungated regions compared to the top gate area, these ungated regions dominate the measured resistance. This effect becomes particularly problematic near the charge neutrality point of the gated region—where precise data is required to extract D_0—as the resistance is dominated by the ungated regions. As a result, an exact value of D_0 could not be determined and is therefore set to $D_0 = 0$.

Hence, while the data points within each sample follow a reproducible trend, points from different samples that are labeled with the same displacement field may correspond to slightly different actual displacement values, due to possible sample-to-sample variations in D_0.

Appendix C: Modelling the proximitized BLG channel

To theoretically describe QPCs in proximitized BLG/TMD heterostructures, we work in the frame of the four-band model, including the effects of confinement and proximity-induced SOC. The full Hamiltonian in valley K^ξ with $\xi \pm 1$ reads

$$H^{\xi}_{BLG} + H_Z + H^{\xi}_{SOC}. \tag{C1}$$

The first term [8, 13, 14],

$$H^{\xi}_{BLG} = \sigma_0 \otimes \xi \begin{pmatrix} \xi U(x) - \frac{1}{2}\Delta(x) & v_3\pi & 0 & v\pi^\dagger \\ v_3\pi^\dagger & \xi U(x) + \frac{1}{2}\Delta(x) & v\pi & 0 \\ 0 & v\pi^\dagger & \xi U(x) + \frac{1}{2}\Delta(x) & \xi\gamma_1 \\ v\pi & 0 & \xi\gamma_1 & \xi U(x) - \frac{1}{2}\Delta(x) \end{pmatrix}, \tag{C2}$$

captures the confinement by a confinement potential U(x) and spatially modulated gap $\Delta(x)$,

$$U(x) = \frac{U_0}{\cosh\frac{x}{L}}, \qquad \Delta(x) = \Delta_0 - \beta\frac{\Delta_0}{\cosh\frac{x}{L}}, \tag{C3}$$

with $\beta = 0.2$ and confinement depth $U_0 = -20$ meV, and width L chosen to match the parameters of the experimental probes. Similar, smooth potential landscapes have been used previously to successfully describe electrostatically confined channels in BLG [8, 10, 14]. Furthermore, $\pi = p_x + ip_y$, $\pi^\dagger = p_x - ip_y$, with $\mathbf{p} = -i\hbar\nabla - \frac{e}{c}\mathbf{A}$, $v = 1.0228 * 10^6$ m/s, $v_3 = 1.2299 * 10^5$ m/s, and $\gamma_1 = 381$ meV.

The second term, $H_Z = -\frac{1}{2}g_S\mu_B B\sigma_z \otimes \sigma_0 \otimes \sigma_0$, with Bohr magneton μ_B and spin g-factor $g_S = 2$, describes spin Zeeman coupling.

The last term describes the proximity-induced SOC [15],

$$H^{\xi=+1}_{SOC} = \sigma_z \otimes \begin{pmatrix} -\lambda_I^{A_1} & 0 & 0 & 0 \\ 0 & \lambda_I^{B_2} & 0 & 0 \\ 0 & 0 & -\lambda_I^{A_2} & 0 \\ 0 & 0 & 0 & \lambda_I^{B_1} \end{pmatrix} \tag{C4}$$

$$+ \sigma_x \otimes \begin{pmatrix} 0 & 0 & 0 & -i\lambda_{R_1}(1-s) \\ 0 & 0 & i\lambda_{R_2}(1+s) & 0 \\ 0 & -i\lambda_{R_2}(1-s) & 0 & 0 \\ i\lambda_{R_1}(1+s) & 0 & 0 & 0 \end{pmatrix}, \tag{C5}$$

and

$$H^{\xi=-1}_{SOC} = \sigma_z \otimes \begin{pmatrix} -\lambda_I^{B_2} & 0 & 0 & 0 \\ 0 & \lambda_I^{A_1} & 0 & 0 \\ 0 & 0 & -\lambda_I^{B_1} & 0 \\ 0 & 0 & 0 & \lambda_I^{A_2} \end{pmatrix} \tag{C6}$$

$$+ \sigma_x \otimes \begin{pmatrix} 0 & 0 & 0 & i\lambda_{R_2}(1-s) \\ 0 & 0 & -i\lambda_{R_1}(1+s) & 0 \\ 0 & i\lambda_{R_1}(1-s) & 0 & 0 \\ -i\lambda_{R_2}(1+s) & 0 & 0 & 0 \end{pmatrix}. \tag{C7}$$

In (C5), (C7), λ_I measures the strength of the intrinsic SOC and λ_R of Rashba SOC, respectively, and $s = \pm 1$ labels the spin states $\uparrow = 1$ and $\downarrow = -1$. We work in the basis $\Phi_{K+} = (\Psi_{A1}\uparrow, \Psi_{B2}\uparrow, \Psi_{A2}\uparrow, \Psi_{B1}\uparrow, \Psi_{A1}\downarrow, \Psi_{B2}\downarrow, \Psi_{A2}\downarrow, \Psi_{B1}\downarrow)$ or $\Phi_{K-} = (\Psi_{B2}\uparrow, \Psi_{A1}\uparrow, \Psi_{B1}\uparrow, \Psi_{A2}\uparrow, \Psi_{B2}\downarrow, \Psi_{A1}\downarrow, \Psi_{B1}\downarrow, \Psi_{A2}\downarrow)$. Note that our convention of labeling the graphene sublattices and layers differs from that of previous works, and to reach consistency with the proximity-induced SOC parameters presented in Refs. [15, 16] one has to interchange sublattices A and B as well as graphene layers 1 and 2. The spin–valley–Zeeman SOC parameter is related to the parameters above as [3]

$$\lambda_{VZ} = \frac{\lambda_I^B - \lambda_I^A}{2}. \tag{C8}$$

To calculate the confined subband spectra and states (as the ones shown in Fig. 3f and Figs. 4c,d of the main text), we numerically diagonalize the Hamiltonian in (C2) in a suitable basis of confined states. We follow the procedure described in Refs. [8, 14], using harmonic oscillator wave functions in the confinement direction across the channel axis, $\tilde{x} = x\sin\theta + y\cos\theta$ and assuming free propagation of the electrons along the channel axis, $\tilde{y} = x\cos\theta - y\sin\theta$, where the angle θ interpolates between orientation of the channel axis along the armchair ($\theta = 0$) and zigzag ($\theta = \frac{\pi}{2}$) direction of the graphene lattice. We refer the reader to Refs. [8, 14] for details about the numerical implementation.

We extract the subband edges for different magnetic field values for deducing the magnetic field patterns as shown in Fig. 4. These points correspond to the energies where we expect steps in the quantized conductance.

Appendix D: Impact of the SOC parameters on the calculated spectrum

We discuss how choosing different SOC parameters in the theoretical model changes the calculated magnetic field patterns compared to experimental observations. In Figs. D.3 and D.4, we show the magnetic field patterns obtained from calculations with different SOC parameters, $\lambda_I^{A_1}, \lambda_I^{A_2}, \lambda_I^{B_1}, \lambda_I^{B_2}, \lambda_{R_1}, \lambda_{R_2}$, ranging from no SOC to strong intrinsic or Rashba SOC, respectively. This analysis allows the following conclusions about the role

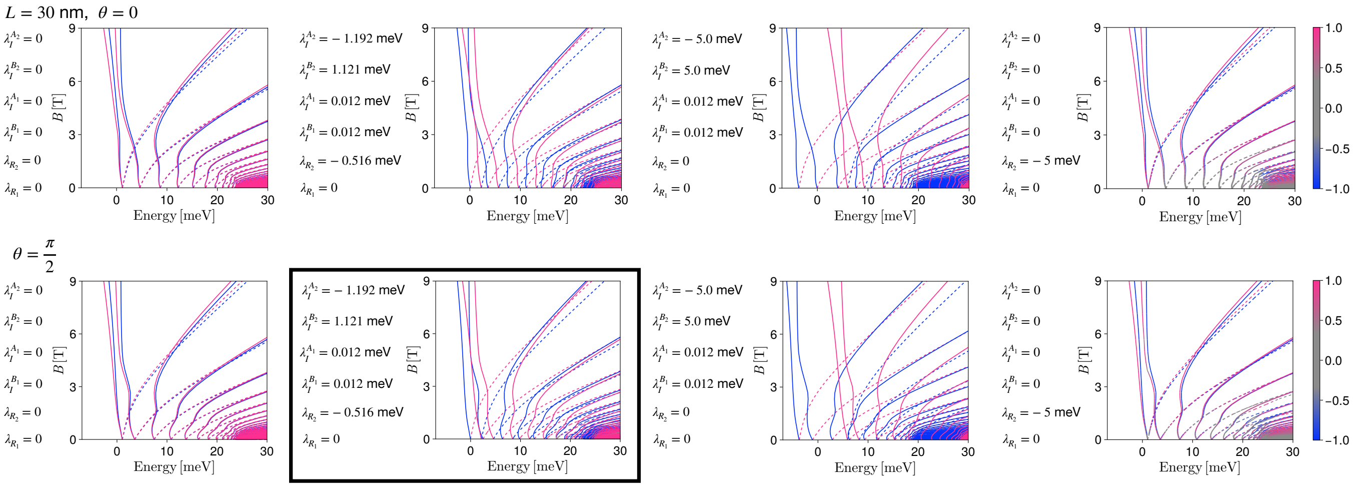

FIG. D.3. Calculated magnetic field patterns for different SOC parameters and channel orientations for width $L = 30$ nm and $\Delta = 50$ meV. The boxed plot corresponds to the one shown in the main text, with parameters obtained from DFT in [16], showing the best agreement with experimental data. Solid and dashed lines distinguish between the K^+ and K^- valley. The color scale quantifies the spin polarization of the bands.

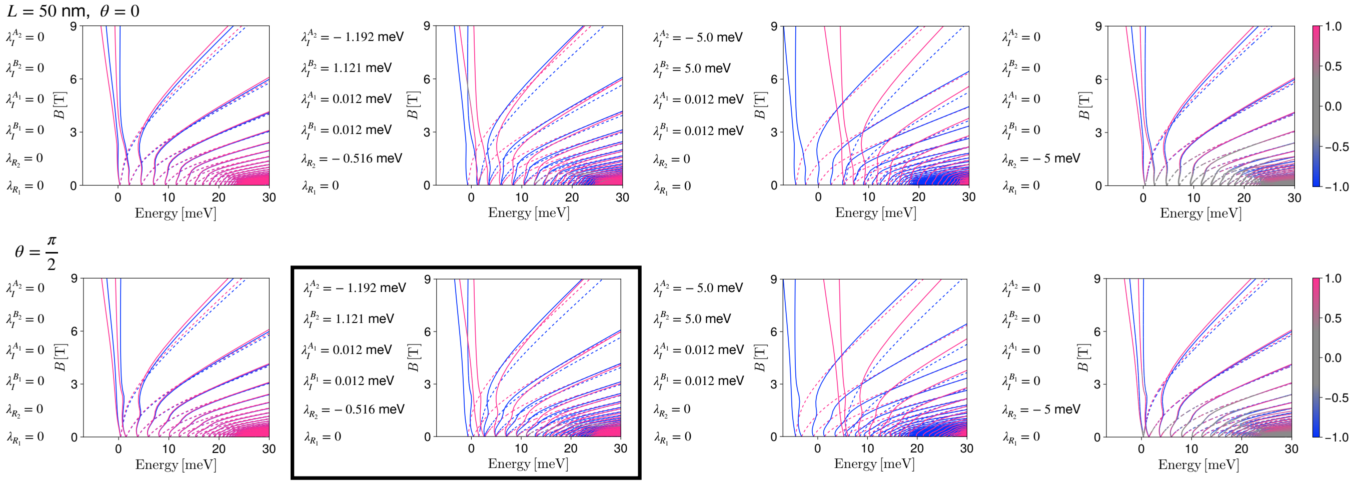

FIG. D.4. Calculated magnetic field patterns for different SOC parameters and channel orientations for width $L = 50$ nm and $\Delta = 50$ meV. The boxed plot corresponds to the one shown in the main text, with parameters obtained from DFT in [16], showing the best agreement with experimental data. Solid and dashed lines distinguish between the K^+ and K^- valley. The color scale quantifies the spin polarization of the bands.

of the SOC parameters: First, we observe that the intrinsic SOC λ_I is indeed responsible for the characteristic 3+1 pattern observed in the experiment (compare first and second column in Figs. D.3 and D.4). However, the value of λ_I cannot be much larger than the subband spacing to obtain this pattern (compare columns two and three). Further, the Rashba parameters have negligible influence on the magnetic field patterns since it does not induce noticeable splittings at the band minima near the K-points, even for large values of λ_R (fourth column in Figs. D.3 and D.4). We note that Rashba SOC does, however, affect the spin polarization of the subbands. In the case of the relatively small value $\lambda_{R_2} = -0.5$ meV predicted by DFT [16], we obtain spin polarizations above 0.99 for all data points (data presented in the main text, Fig. 3). Increasing λ_{R_2} decreases the degree of spin polarization. For $\lambda_{R_2} = -5$ meV in the fourth column of Figs. D.3 and D.4, we find the spin polarizations vary significantly depending on the band index and the strength of the external magnetic field.

Appendix E: Additional measurement data

1. PNP dot with weak spin-orbit coupling

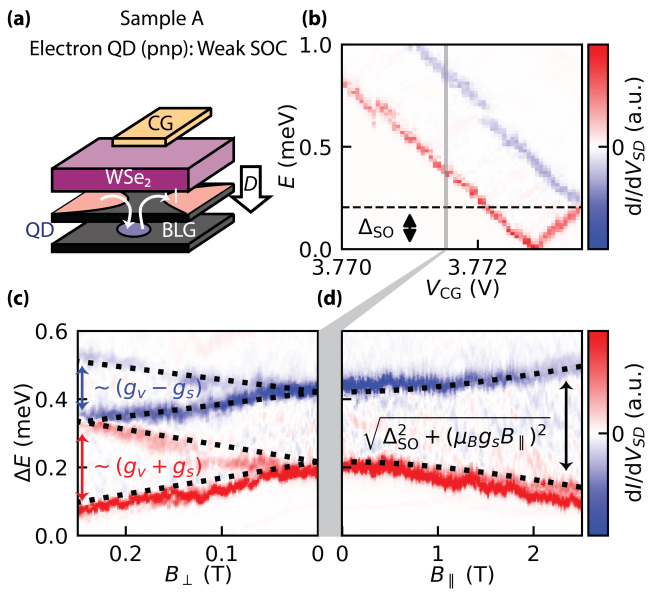

FIG. E.5. **(a)** Schematic of the pnp-type quantum dot in sample A (WSe_2/BLG). **(b)** Finite-bias spectroscopy of the first charge carrier at $B_\perp = 0$ and $T = 10\,\text{mK}$. **(c)**, **(d)** Evolution of the QD energy levels in perpendicular and parallel magnetic fields, respectively. The data are overlaid with theoretical curves calculated using the extracted spin–orbit splitting Δ_{SO}, valley g-factor $g_v = 14.4$, and spin g-factor $g_s = 2$. The energy levels were extracted from bias spectroscopy performed at varying magnetic fields, as indicated by the gray line.

To clearly demonstrate the expected quadratic dependence on magnetic field, we turn to measurements in the weak SOC regime, conducted on the pnp-type quantum dot in sample A (WSe_2/BLG), shown in Fig. E.5a. Finite-bias spectroscopy (Fig. E.5b) yields a spin–orbit splitting of Δ_{SO} =(0.21 ± 0.02) meV. Measurements in a perpendicular magnetic field reveal a clear splitting of each Kramers pair, consistent with the valley–Zeeman effect (Fig. E.5c). By analyzing the energy separation within each pair—given by $(g_v + g_s)\mu_B B_\perp$ and $(g_v - g_s)\mu_B B_\perp$—we extract valley and spin g-factors of g_v =(14.4 ± 0.3) and g_s =(2.0 ± 0.3) , respectively. The data are overlaid with the theoretically expected evolution in $B_\perp$ (dotted line), calculated using these g-factors and the zero-field spin–orbit splitting $\Delta_{SO} = 0.21\,\text{meV}$ from Fig. E.5b. The agreement between theory and experiment confirms that each Kramers pair comprises two states with opposite valley and spin quantum numbers.

Compared to the quantum dot presented in Fig. 2, the reduced SOC in this regime allows for a more unambiguous observation of the expected quadratic dependence of the energy splitting on $B_\parallel$. This behavior is clearly visible in Fig. E.5d, confirming that the two Kramers pairs arise from opposite spin orientations coupled via an out-of-plane spin–orbit field. The data are overlaid with the theoretically expected evolution, calculated using the extracted spin–orbit gap Δ_{SO} and the spin g-factor $g_s = 2$. The slight deviation from the quadratic trend at higher fields is attributed to a small $B_\perp$ component resulting from the residual tilt of the sample.

Overall, the measurements closely follow the expected quadratic evolution of the spin–orbit gap with in-plane magnetic field. In contrast, an orbital excited state would exhibit a $B_\parallel$ dependence identical to the ground state, which shares the same spin configuration. The distinct responses to both in-plane and out-of-plane magnetic fields therefore provide unambiguous evidence that the measured excitation corresponds to the spin–orbit splitting of the first charge carrier. The observed magnetic field dependence is fully consistent with previous studies on pristine bilayer graphene QDs [17–19], with the notable distinction of a significantly enhanced spin–orbit gap in the present devices.

2. MoS_2/BLG reference QPC

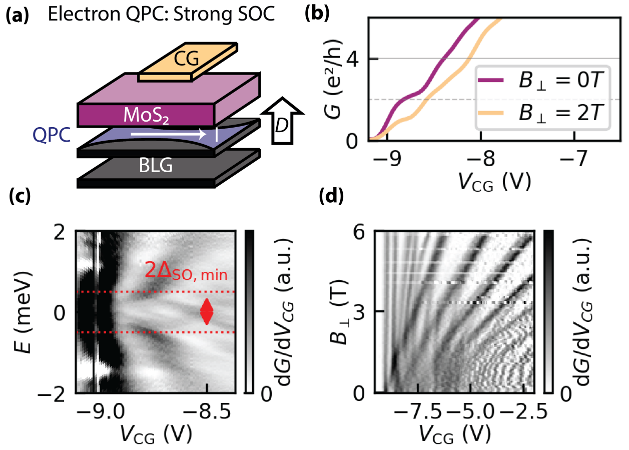

FIG. E.6. **(a)** Schematic side view of the MoS_2/BLG device. **(b)** Four-terminal conductance traces at $B_\perp = 0\,\text{T}$ and $B_\perp = 2\,\text{T}$. **(c)** Bias spectroscopy of the $G = 2e^2/h$-step, indicating a $\Delta_{SO} > 500\,\mu\text{eV}$. **(d)** Transconductance as function of $B_\perp$ and V_{CG}.

We demonstrate that replacing WSe_2 with MoS_2 in the same device geometry (Fig. E.6a) leads to a similar enhanced and tunable SOC. We measure a clear $G = 2e^2/h$ plateau in Fig. E.6b (uncorrected conductance), indicating a lifted degeneracy, caused by an enhanced SOC. This plateau further splits at a relatively small $B_\perp$ due to the valley–Zeeman effect (Fig. E.6b). Fig. E.6c presents the energy gap between the two lowest states. Due to a low subband spacing in the measured QPC, direct state assignment in the $B_\perp$ transconductance data is not possi-

ble (Fig. E.6d). Thus, we interpret the measured gap of 500 µeV as a lower bound for Δ_{SO}. Measurements were taken at $T = 10\,\mathrm{mK}$.

3. Bilayer graphene reference QPC

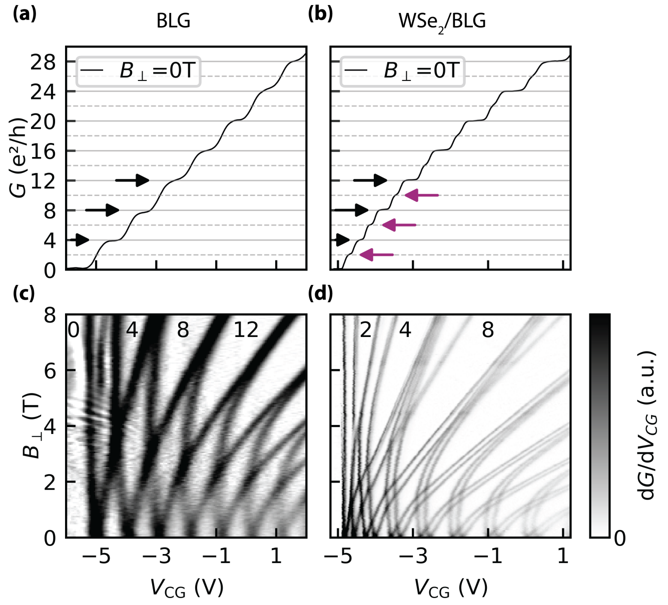

FIG. E.7. **(a), (b)** Four-terminal conductance G as a function of the channel gate voltage V_{CG} (T = 1.3 K) for BLG **(a)** and WSe_2/BLG **(b)**. The traces have been corrected from parasitic resistances.**(c), (d)** Two-terminal transconductance dG/dV_{CG} as function of V_{CG} and $B_\perp$ at T = 1.3 K for BLG **(c)** and WSe_2/BLG **(d)**. The black numbers in the plot represent the quantum numbers of the respective conductance plateaus.

In this section, we present the magnetic field-dependent transconductance of the reference BLG QPC, whose conductance trace was shown in Fig. 1c, and compare it to the corresponding measurements for the WSe_2/BLG QPC. The BLG QPC has a 100 nm channel width - in contrast to the 75 nm channel width used for all other QPCs in this study.

Figures E.7a,b display the corrected conductance plateaus of the BLG and WSe_2/BLG QPC, while Figs. E.7c,d show the respective transconductance dG/dV_{CG} as a function of $B_\perp$. In the pristine BLG QPC, increasing $B_\perp$ splits the subbands due to the valley–Zeeman effect, forming two valley-polarized pairs: ($K^+\downarrow$, $K^+\uparrow$) and ($K^-\uparrow$, $K^-\downarrow$) (Fig. 1c). At sufficiently high magnetic fields, the magnetic length becomes smaller than the channel width, leading to the formation of Landau levels and an apparent convergence of the $(n+2)K^+$ and nK^- states. Similar behavior in BLG QPCs has been reported in Refs. [6–10].

The magnetotransport data for the WSe_2/BLG QPC (Fig. E.7d) strongly resembles that of pristine BLG (Fig. E.7c), with one crucial distinction: in BLG/WSe_2, two parallel evolving states within the same subband and same valley quantum number remain well-resolved due to the enhanced spin–orbit splitting. In contrast, in pristine BLG, these states are too close in energy to be resolved separately.

Additionally, we note that the quality of the pristine BLG QPC device appears to be lower compared to the WSe_2/BLG QPC. While we do not attribute this to a fundamental effect, this observation suggests that introducing a TMD layer does not reduce device quality more than typical sample-to-sample variations in BLG-based quantum devices.

4. Spin–orbit gap measurements across all device configurations

In the following table (Fig. E.8), we present representative bias spectroscopy measurements for extracting the spin–orbit gaps Δ_{SO} across all eight possible configurations. The measured values show excellent agreement with layer polarization predictions, which are also included in the figure.

For the p-type QPCs (Fig. E.8b, d), an exact Δ_{SO} could not be determined, as the subband spacing of the lowest modes is smaller than the spin–orbit gap, making an unambiguous assignment of low-energy subband states at zero magnetic field impossible. However, strong SOC is evident from the highly broken degeneracy. The magenta circle highlights the states $3K^+\downarrow$ and $3K^+\uparrow$, which originate from different energies at $B = 0\,\mathrm{T}$ in the high-SOC case (Fig. E.8b), whereas in the low-SOC scenario (Fig. E.8d), they originate from nearly the same zero-magnetic-field energy.

5. State Evolution in a Strong SOC Quantum Dot under Magnetic Fields

In this chapter, we present magnetic field measurements corresponding to the high SOC quantum dot in sample B (WSe_2/BLG), whose results were shown in Fig. 2. Since the excited state energies of interest are relatively high, we cut the Coulomb diamond at a constant energy of $E = 1.9\,\mathrm{meV}$ and sweep the V_{CG}-axis in a magnetic field. The measurements are overlaid with the expected behavior using the extracted lever arm.

From the $B_\perp$ data, it is evident that the current through the quantum dot is strongly suppressed above $B_\perp = 0.15\,\mathrm{T}$, reaching the noise level. As a result, a more precise extraction of the valley g_v-factor was not possible.

The $B_\parallel$ data, on the other hand, clearly follows the expected trend. However, a quadratic dependence is not distinctly resolved due to the large SOC.

Overall, the states follow the single-particle predictions well.

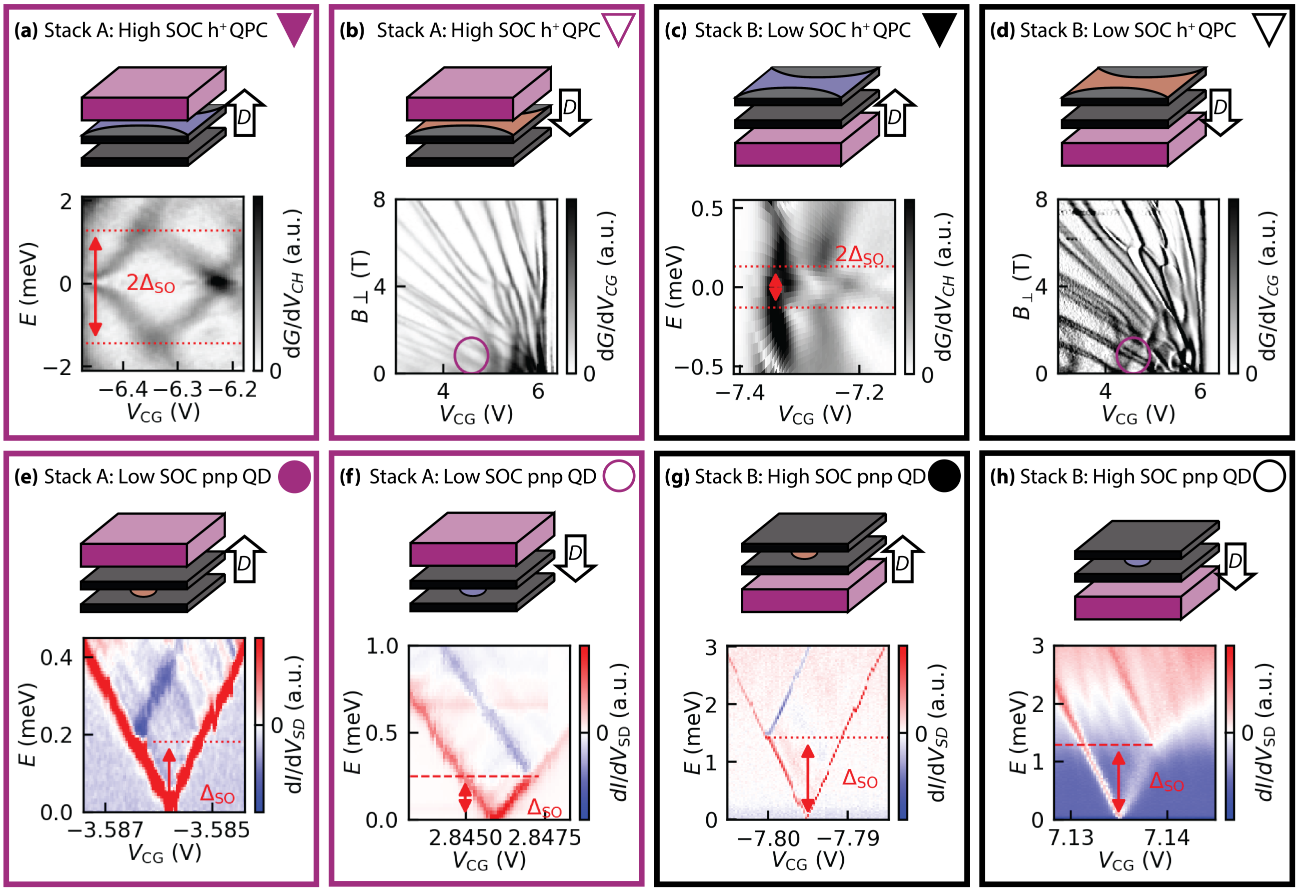

FIG. E.8. Exemplary bias spectroscopy across all configurations at 10 mK, showing the extracted spin–orbit gap Δ_{SO} and a schematic electrostatic illustration. For the p-type QPC in both samples, Δ_{SO} could not be determined due to a low subband spacing. We instead present magneto transconductance data for **(b)** and **(d)** verifying the expected SOC trend qualitatively when comparing the $3K^+\downarrow$ and $3K^+\uparrow$ states in this system highlighted by the magenta circle.

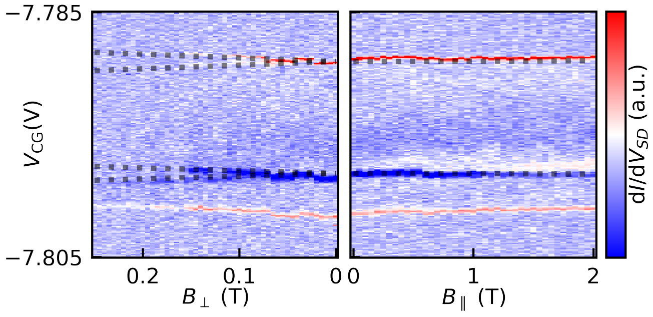

FIG. E.9. Magnetic field evolution of the quantum dot states in sample B (as shown in Fig. 2), measured in both $B_\perp$ (left) and $B_\parallel$ (right). The state evolution is overlaid with a gray dotted line, showing the expected behavior based on the extracted values Δ_{SO}, $g_v \approx 14$, and $g_s = 2$.

6. High magnetic field transport data of high-SOC QPCs at 10 mK

In this section, we present the high-magnetic-field transconductance data from Figs. 4a,b, and compare it with theoretical predictions. The corresponding band structures for channel widths of 30 nm and 50 nm are shown in Figs. E.10a,b. Figs. E.10c,d depict the single-particle calculations, which qualitatively match the measured magnetotransport data in Figs. E.10e,f. By analyzing the measured magnetic field dependence, we can confidently assign all observed states in the measurements, confirming that the subband spacing in Fig. E.10f is smaller than in Fig. E.10e.

The 10 mK measurement temperature improves the resolution of each subband state. Both experimental data and theoretical predictions consistently show that the previously observed 3+1 feature evolves into a “2+1+1” splitting at higher magnetic fields. Additionally, we observe that in a high magnetic field the $2K^-\uparrow$ splits off from the $2K^-\downarrow/4K^+$ branch in a similar way to the $1K^-\uparrow$ state from $1K^-\downarrow/3K^+$. This increased mag-

netic field for the second split-off is also captured in the model. Furthermore, the model correctly predicts the trend, that the magnetic split-off field decreases for configurations with lower subband spacing.

This "3+1" feature enables an unambiguous assignment of all observed quantum states. This characteristic feature, evident in both experiment and theory, arises from the energetic separation of the $1K^- \uparrow$ state from the closely spaced $1K^- \downarrow/3K^+$ branch. This pattern results from the interplay between SOC and the spin–Zeeman effect in opposite valleys. In the absence of SOC, the states would organize into two Zeeman-split pairs, as shown in SI D Figs. D.3 and D.4. However, the presence of strong SOC induces opposite spin splittings in opposite valleys. For the $1K^-$ states, SOC and the spin–Zeeman effect act in the same direction, thereby enhancing the energy separation between $1K^- \uparrow$ and $1K^- \downarrow$, leading to a pronounced spin split-off state at high magnetic field. In contrast, for the $3K^+$ states, SOC counteracts the spin–Zeeman effect, which reduces the energy gap between $3K^+ \uparrow$ and $3K^+ \downarrow$ as the field increases—ultimately giving rise to the observed 3+1 state pattern. The observation of this feature in both calculated and measured magnetic field spectra provides compelling evidence for opposite spin orientations in states associated with the same subband and valley index. The appearance of this feature in higher branches allows for a recursive assignment of quantum numbers to all observed states. By tracing the K^- and K^+ states back to zero magnetic field, we identify the ground states as $1K^+ \uparrow/1K^- \downarrow$, and the excited states as $1K^- \uparrow/1K^+ \downarrow$, in agreement with theoretical calculations. The energy gap between these states thus corresponds to the spin–orbit gap, characterized by spin–valley–Zeeman SOC.

Overall, the strong agreement between theory and experiment highlights the model's robustness and a strong understanding of this system.

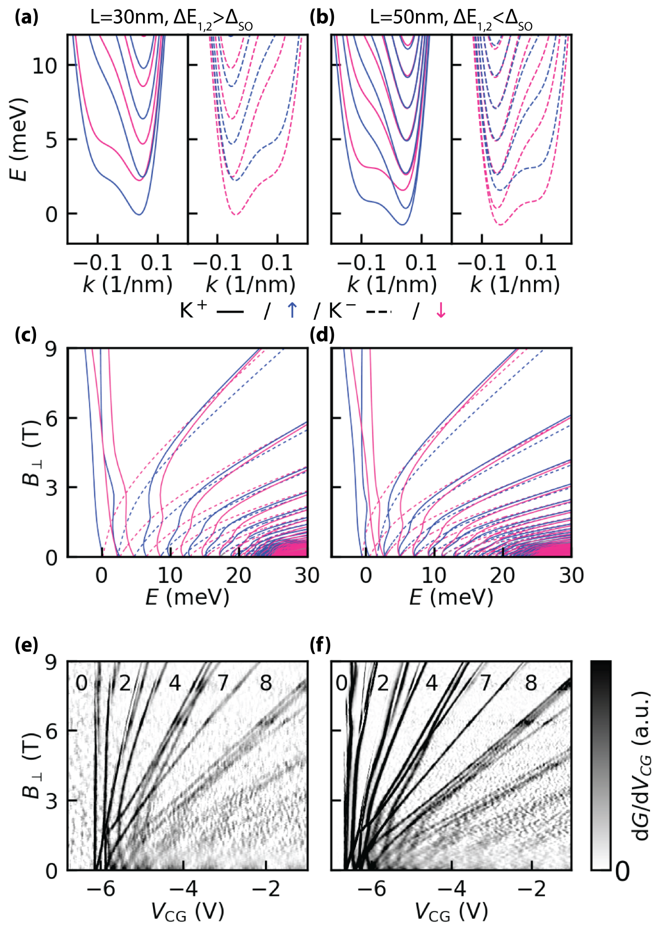

FIG. E.10. **(a)**, **(b)** Calculated band structure for WSe_2/BLG QPCs with channel widths of 30 nm and 50 nm, respectively. **(c)**, **(d)** Corresponding single-particle calculations of subband evolution in a perpendicular magnetic field. **(e)**, **(f)** Experimentally measured magnetotransport data at high magnetic fields and 10 mK, with zoomed-in sections shown in Fig. 4a, b. Although both QPCs have the same 75 nm lithographic width, they exhibit different subband spacings, qualitatively matching the single-particle calculations in **(c)** and **(d)**. Possible explanations for this behavior are discussed in the main text.

[1] A. David, P. Rakyta, A. Kormányos, and G. Burkard, Induced spin-orbit coupling in twisted graphene–transition metal dichalcogenide heterobilayers: Twistronics meets spintronics, Phys. Rev. B **100**, 085412 (2019).

[2] Y. Li and M. Koshino, Twist-angle dependence of the proximity spin-orbit coupling in graphene on transition-metal dichalcogenides, Phys. Rev. B **99**, 075438 (2019).

[3] T. Naimer, K. Zollner, M. Gmitra, and J. Fabian, Twist-angle dependent proximity induced spin-orbit coupling in graphene/transition metal dichalcogenide heterostructures, Phys. Rev. B **104**, 195156 (2021).

[4] K. Zollner, S. a. M. João, B. K. Nikolić, and J. Fabian, Twist- and gate-tunable proximity spin-orbit coupling, spin relaxation anisotropy, and charge-to-spin conversion in heterostructures of graphene and transition metal dichalcogenides, Phys. Rev. B **108**, 235166 (2023).

[5] Y. Zhang, G. Shavit, H. Ma, Y. Han, C. W. Siu, A. Mukherjee, K. Watanabe, T. Taniguchi, D. Hsieh, C. Lewandowski, F. von Oppen, Y. Oreg, and S. Nadj-Perge, Twist-programmable superconductivity in spin–orbit-coupled bilayer graphene, Nature **641**, 625 (2025).

[6] R. Kraft, I. V. Krainov, V. Gall, A. P. Dmitriev, R. Krupke, I. V. Gornyi, and R. Danneau, Valley subband splitting in bilayer graphene quantum point contacts, Phys. Rev. Lett. **121**, 257703 (2018).

[7] H. Overweg, H. Eggimann, X. Chen, S. Slizovskiy, M. Eich, R. Pisoni, Y. Lee, P. Rickhaus, K. Watanabe, T. Taniguchi, V. Fal'ko, T. Ihn, and K. Ensslin, Electrostatically induced quantum point contacts in bilayer graphene, Nano Letters **18**, 553 (2018).

[8] H. Overweg, A. Knothe, T. Fabian, L. Linhart, P. Rickhaus, L. Wernli, K. Watanabe, T. Taniguchi, D. Sánchez, J. Burgdörfer, F. Libisch, V. I. Fal'ko, K. Ensslin, and T. Ihn, Topologically nontrivial valley states in bilayer graphene quantum point contacts, Phys. Rev. Lett. **121**, 257702 (2018).

[9] L. Banszerus, B. Frohn, T. Fabian, S. Somanchi, A. Epping, M. Müller, D. Neumaier, K. Watanabe, T. Taniguchi, F. Libisch, B. Beschoten, F. Hassler, and C. Stampfer, Observation of the spin-orbit gap in bilayer graphene by one-dimensional ballistic transport, Phys. Rev. Lett. **124**, 177701 (2020).

[10] Y. Lee, A. Knothe, H. Overweg, M. Eich, C. Gold, A. Kurzmann, V. Klasovika, T. Taniguchi, K. Wantanabe, V. Fal'ko, T. Ihn, K. Ensslin, and P. Rickhaus, Tunable valley splitting due to topological orbital magnetic moment in bilayer graphene quantum point contacts, Phys. Rev. Lett. **124**, 126802 (2020).

[11] M. Masseroni, Electronic transport experiments in 2d materials with spin-orbit coupling, Doctoral Thesis ETH (2024).

[12] Y. Hou, G. Wang, C. Ma, Z. Feng, Y. Chen, and T. Filleter, Quantification of the dielectric constant of MoS_2 and WSe_2 nanosheets by electrostatic force microscopy, Materials Characterization **193**, 112313 (2022).

[13] E. McCann and M. Koshino, The electronic properties of bilayer graphene, Reports on Progress in Physics **76**, 056503 (2013).

[14] A. Knothe and V. Fal'ko, Influence of minivalleys and berry curvature on electrostatically induced quantum wires in gapped bilayer graphene, Phys. Rev. B **98**, 155435 (2018).

[15] K. Zollner and J. Fabian, Bilayer graphene encapsulated within monolayers of WS_2 or $Cr_2Ge_2Te_6$: Tunable proximity spin-orbit or exchange coupling, Phys. Rev. B **104**, 075126 (2021).

[16] A. M. Seiler, Y. Zhumagulov, K. Zollner, C. Yoon, D. Urbaniak, F. R. Geisenhof, K. Watanabe, T. Taniguchi, J. Fabian, F. Zhang, and R. T. Weitz, Layer-selective spin-orbit coupling and strong correlation in bilayer graphene, 2D Materials **12**, 035009 (2025).

[17] L. Banszerus, S. Möller, C. Steiner, E. Icking, S. Trellenkamp, F. Lentz, K. Watanabe, T. Taniguchi, C. Volk, and C. Stampfer, Spin-valley coupling in single-electron bilayer graphene quantum dots, Nature Communications **12**, 5250 (2021).

[18] A. Kurzmann, Y. Kleeorin, C. Tong, R. Garreis, A. Knothe, M. Eich, C. Mittag, C. Gold, F. K. de Vries, K. Watanabe, T. Taniguchi, V. Fal'ko, Y. Meir, T. Ihn, and K. Ensslin, Kondo effect and spin–orbit coupling in graphene quantum dots, Nature Communications **12**, 6004 (2021).

[19] H. Duprez, S. Cances, A. Omahen, M. Masseroni, M. J. Ruckriegel, C. Adam, C. Tong, R. Garreis, J. D. Gerber, W. Huang, L. Gächter, K. Watanabe, T. Taniguchi, T. Ihn, and K. Ensslin, Spin-valley locked excited states spectroscopy in a one-particle bilayer graphene quantum dot, Nature Communications **15**, 9717 (2024).
